# The long-term impact of restricted access to abortion on children’s socioeconomic outcomes

**DOI:** 10.1371/journal.pone.0248638

**Published:** 2021-03-15

**Authors:** Gábor Hajdu, Tamás Hajdu

**Affiliations:** 1 Institute for Sociology, Centre for Social Sciences, Budapest, Hungary; 2 Institute of Economics, Centre for Economic and Regional Studies, Budapest, Hungary; University of Copenhagen, DENMARK

## Abstract

We examine the long-term consequences of restricted access to abortion following a change in the Hungarian abortion law in 1974. Due to a change that restricted access to legal abortions, the number of induced abortions decreased from 169,650 to 102,022 between 1973 and 1974, whereas the number of live births increased from 156,224 to 186,288. We analyze the effects on the adult outcomes of the affected cohort of newborns (educational attainment, labor market participation, teen fertility). We use matched large-scale, individual-level administrative datasets of the Hungarian Central Statistical Office (population census 2011; live birth register), and we estimate the effects by comparing children born within a short timespan around the time the law change came into effect. We apply a difference-in-differences approach, building on the special rules of the new law that, despite the severe restriction, still made abortion permissible for selected groups of women. We control for the compositional change in the population of parents, rule out the effect of (unobserved) time trends and other potential behavioral responses to the law change, and draw causal inferences. We find that restricted access to abortion had, on average, a negative impact on the socioeconomic outcomes of the affected cohort of children. Children born after the law change have had worse educational outcomes, a greater likelihood of being unemployed at age 37, and a higher probability of being a teen parent.

## 1. Introduction

The impact of access to abortion is an important topic for scientific research. Changes to abortion rules are a continual issue in public debates, and countries worldwide, as well as some states in the USA, are considering restricting or have recently restricted abortion access. There is extensive literature focusing on the effect of abortion on fertility [1–10] and on health at birth [2,11–15]. On the other hand, evidence on the effects of abortion rules on adult outcomes is based on a limited number of countries and abortion law changes. Most of the relevant papers have analyzed the effects of legalizing abortion in the United States. These papers focus on teen childbearing [16,17], educational attainment [18–20], poverty, or earnings [12,18,19]. Further research addresses other outcomes, such as crime [18,21–27] or substance use [28].

Despite the well-documented case of the US, evidence is scarce regarding other countries. For Romania, Pop-Eleches [29] found that children born after the 1966 abortion ban had worse educational and labor market outcomes when the compositional change of the mothers was controlled for. The results were interpreted as the consequences of the ensuing higher number of unplanned, mistimed, or unwanted pregnancies. Mølland [30] shows that after access to abortion in Oslo, Norway was liberalized in the 1960s, children of the mothers who had gained access to abortion had increased education and employment achievements and a reduced use of welfare.

Theoretically, a change in the abortion policy might affect the (average) outcomes of children in the long run through a number of mechanisms [14,19,29]. First, when abortion is less available, the number of unplanned, mistimed, or unwanted pregnancies might increase due to increased costs of abortion (unwantedness effect). This unwantedness effect reflects the direct mechanisms through which restricted access to abortion might have negative effects on children: (i) According to the standard model of child quality-quantity trade-off, an increase in the number of children might negatively affect the living circumstances of all children [31,32]. (ii) It is also possible that restricted access to abortion makes women less able to delay childbearing until a more optimal time when it does not conflict with their educational and labor market plans or with their personal circumstances [33–37]. This potential conflict might cause unfavorable emotional and material conditions for giving birth and raising a child. (iii) Restricted access to abortion might lead to insufficient or delayed prenatal care due to the unwantedness of the pregnancy [38–41]. Second, since cohort size increases after restrictions in abortion rules, a negative crowding effect might emerge [29]. Third, a change in the abortion policy might affect the socioeconomic composition of women carrying pregnancies to term, and this compositional change might influence the average outcomes of children. The direction of this effect is ambiguous both theoretically and empirically. Empirical studies have documented negative effects in the US [12,18,42] and positive effects in Romania and Norway [29,30].

In this research, we focus on the unwantedness effect and examine the long-term consequences of the restrictive Hungarian abortion policy introduced in 1974. We analyze the causal effects of the restrictive abortion policy on the later socioeconomic outcomes of the affected children. We compare children born just before and after the law change came into effect and utilize the fact that the new abortion rules made abortion permissible for specific groups of women. We use matched large-scale, individual-level administrative datasets (live birth registry and the 2011 census) and apply a difference-in-difference approach, controlling for a rich set of parental sociodemographic characteristics at the time of birth. Using this empirical approach, the compositional change of the parents and the crowding effect are controlled for; thus, we measure the unwantedness effect.

We find that the restrictive Hungarian abortion policy had, on average, negative long-term impacts on the affected children. Compared to children born just before the restriction, children born after the law change have had worse educational outcomes (e.g., fewer years of education), a greater likelihood of being unemployed at age 37 and a higher probability of having been a teen parent.

This paper contributes to the literature in several ways. First, as we noted, there are only a few papers that analyze the long-term impact of abortion restrictions on affected children outside the US. Since changes in abortion laws are rare, any evidence about the impacts of previously uninvestigated legal changes offers important insights and helps to obtain a more complete picture of the consequences of access to abortion. For example, access to abortion was restricted in Romania in 1966, and there were extreme regulations that made abortion and family planning illegal for almost every woman. The change in Hungarian law in 1974 was less extreme; thus, our paper provides information about how a less drastic policy change affects the socioeconomic outcomes of children. Additionally, we do not know of other papers that analyze the impact of abortion restrictions (rather than the impact of legalization) on the long-term outcomes of children. Second, most of the previous papers, except those of Pop-Eleches [29] and Lin and Pantano [19], have been unable to distinguish the mechanisms through which changes in abortion law affect the outcomes of children. Here, we focus on one specific mechanism: the consequences of the increased number of children from unwanted pregnancies due to the new restrictions. Finally, we use individual-level registry data and apply a difference-in-difference strategy that is rare in the literature.

The paper is structured as follows. First, the law change is introduced (Section 2). Section 3 presents the data and empirical strategy. Section 4 shows the results and the robustness tests. Section 5 discusses the limitations of the study, and Section 6 concludes the paper.

## 2. Background

In the second half of the 1950s, Hungarian abortion rules could be considered liberal. Abortion was allowed on demand [43] and it was a standard method of family planning and contraception [44]. Although women had to submit their requests for abortion to abortion committees, these committees approved the request if the woman reaffirmed the request after receiving information about the abortion [44].

On January 1, 1974, new, restricted abortion rules were introduced. Formally, the rules were justified as intending to protect women’s health, but the real goal was to reduce the high number of abortions and increase fertility [43]. The most important change was that access to abortion was restricted to specific groups: unmarried women, women with three or more children, women over the age of 35, women with serious housing problems or living in poverty, and cases when pregnancy would cause serious health hazards for the mother [43–45]. Moreover, women seeking abortion for nonmedical reasons were charged a substantial fee that was 20–35% of the average gross monthly earnings of employees [44,46]. In each case, abortion committees decided whether to grant the abortion request. These committees consisted of one doctor, one visiting healthcare professional, and one-three lay members who were chosen by the head of the health department of the district soviet [44]. Applications had to be submitted to the abortion committees in person, and women requesting abortion had to be present when the committee made a decision on their request. This application procedure was humiliating and corrupt, even for women who had a good chance of a positive decision [43,45,46]. Additionally, a media campaign attacked abortion (and birth control methods) as “unacceptable in a socialist society” since it was rooted in the “‘unhealthy’ spirit of individualism” [43].

**Fig 1:**
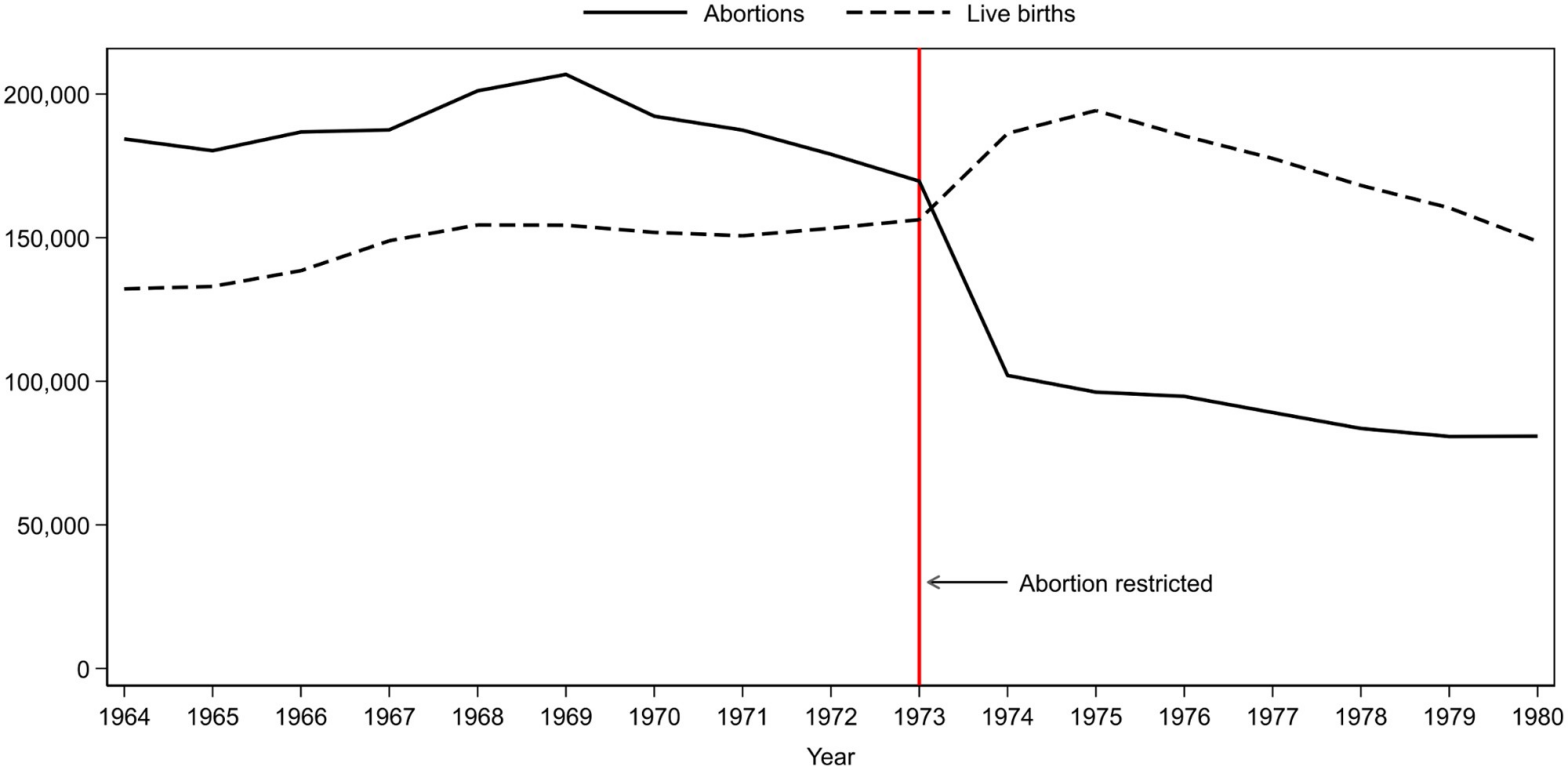
Number of induced abortions and live births between 1964 and 1980. Source: Hungarian Central Statistical Office (http://www.ksh.hu/docs/eng/xstadat/xstadat_long/h_wdsd001a.html and http://www.ksh.hu/docs/eng/xstadat/xstadat_long/h_wdsd001b.html).

Fig 1 shows that the law change had a substantial effect on the number of live births and the number of induced abortions. Between 1973 and 1974, the number of induced abortions decreased from 169,650 to 102,022, and the number of live births increased by 30,000 (from 156,224 to 186,288). In other words, the number of induced abortions per 100 live births decreased by 50%, dropping from 108.6 to 54.8. On the other hand, the decrease in the number of induced abortions was twice as large as the increase in the number of births. This might reflect a relatively quick adaptation to the new rules. Although the governmental decision about the changes was made in October, as early as summer, information on the planned changes to the law was available; therefore, women were not entirely “surprised” by the policy change. There is also indirect evidence that suggests that illegal or semi-illegal abortions that were not included in the official statistics might have supplemented the number of legal abortions [47,48]. Last, access to contraceptives increased in the early 1970s [49], which might also play a role in the adaptation process.

## 3. Methods

### 3.1. Data

We use matched large-scale, individual-level administrative datasets of the Hungarian Central Statistical Office (HCSO) (population census of 2011 and live birth register). No ethical approval was needed for this study because it was based on secondary analysis. The study used completely anonymized data with no identifiable information. We accessed the deidentified datasets in the secure data environment of the HCSO after an accreditation process. The researchers were required to sign a contract and a confidentiality commitment.

The birth register includes every live birth in Hungary since 1970. We link the birth records to the 2011 census to identify long-term outcomes. The variables we use for the linkage were the exact date of birth, the sex of the newborn, and the municipality of residence at the time of birth. The proportion of linked birth records was 34.9% for live births between 1971 and 1979.

This matched dataset contains information on the date of birth, sex of the newborn, characteristics of both parents at the time of birth (i.e., age, education, marital status, employment, occupation code from the standard classification of occupations in Hungary, municipality of residence, and mother’s pregnancy history), and socioeconomic characteristics of the children in 2011. With the exception of socioeconomic characteristics of the children in 2011 (outcome variables), all variables used in our analysis came from the birth records. We use eight outcome variables. First, educational achievement is measured (1) as having a university degree or (2) as having only primary education and (3) by the number of years of education completed. Second, labor market activity is measured by (4) not being employed (according to the ILO definition), (5) working (self-categorization), or (6) being unemployed (self-categorization). We also determine (7) whether the child became a teen parent and, as the only available welfare indicator, (8) whether the child or her/his family own the dwelling where she/he lives.

### 3.2. Empirical strategy

We utilize the fact that the new rules permitted abortions for women who were at least 35 years old. Therefore, we compare children born to mothers who were under age 35 at the time of conception and children born to mothers who were over age 35 at the time of conception. To ensure that the groups are as similar as possible, we use a ±1.5-year time range, and we exclude women who were approximately 35 years old because we have no information about the exact decision-making process of the abortion committees, and we do not know how they evaluated the abortion requests of women near the age limit. Specifically, we use the mother’s age at giving birth because data for her age at the time of conception and for the length of gestation in the birth register are less reliable for the first half of the 1970s. The group of mothers over age 35 at the time of conception consisted of women who were 35.77–37.27 years old at the time of giving birth, and the group of mothers under age 35 at the time of conception consisted of women who were 33.88–35.38 years old at the time of giving birth. The length of most pregnancies is 40 weeks (0.77 years), and abortion was available before the 12^th^ week of pregnancy (0.15 years). Therefore, mothers in the first (“over age 35“) group were at least 35 years old when they conceived, and mothers in the second (“under age 35”) group were 35 years old or younger in the 12^th^ week of pregnancy, even if they gave birth in the 33rd week.

**Fig 2:**
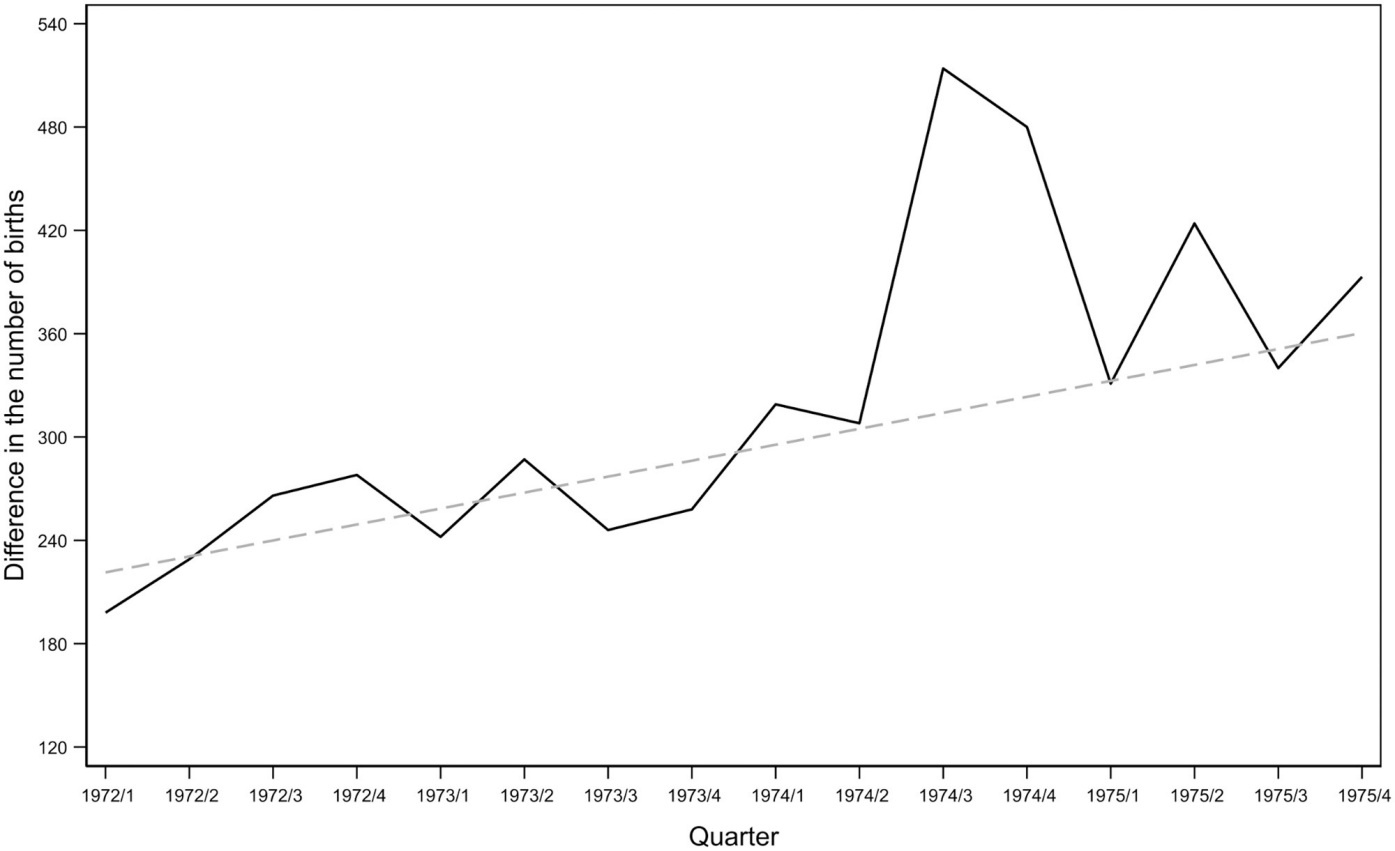
The difference between the number of births among mothers under age 35 and mothers over age 35. The graph shows quarterly values. The solid line shows the difference in the number of births. The dashed line shows the predicted values based on a linear OLS regression using data between 1972/1 and 1974/2. Difference: number of births among mothers under age 35 − number of births among mothers over age 35. Mothers under age 35 at the time of conception were 33.88–35.38 years old when giving birth; mothers over 35 at the time of conception were 35.77–37.27 years old when giving birth.

Fig 2 shows that the difference between the number of births among mothers under age 35 and the number of births among mothers over age 35 increased in the second half of 1974 and then largely returned to previous levels by 1975. This is in line with the fact that restricted abortion rules affected women under age 35 and women over age 35 differently. (In addition, S1 Fig shows the number of births for the two groups of mothers. S1 Table reports the results of regression models using monthly data that show that the increase among mothers under age 35 was significant, whereas there was no relevant change among mothers over age 35. Columns 3 and 4 include data for 1973 and show that the increase cannot be explained by seasonal differences, which are similar across years). Nevertheless, this graph suggests that relatively quick adaptation occurred. This adaptation process may have included the increased use of available legal birth control technologies [49] or resorting to illegal or semi-illegal abortions [47,50]. It is also possible that women became familiar with the decision-making process of the abortion committees and were able to argue their cases convincingly [43,46].

We estimate the effects of the law change by comparing children born just before and after the new law came into effect, which is a similar empirical strategy to those used by Pop-Eleches [21] and Mitrut and Wolff [14]. Specifically, we compare children born between July and September 1974 to children born between April and June 1974, i.e., we compare children whose mothers had full access to abortion and children whose mothers had no access or restricted access to abortion. Using a reasonably short time span, we are able to rule out the effects of other (unobserved) time trends and other potential behavioral responses to the law change, and we can draw causal inferences. The sample size of this analysis is 1124.

Using mothers in these two age groups, we apply a difference-in-differences framework to estimate the causal impact of abortion restrictions on the socioeconomic outcomes of children in 2011 (at approximately age 37). We estimate the following equation:

$$Y_{i}=\beta_{0}+\beta_{1}Under35_{i}+\beta_{2}After_{i}+\beta_{3}Under35_{i}\times After_{i}+\beta_{4}X_{i}+\varepsilon_{i}$$ (1)

where *Y*_*i*_ is an outcome of interest for child *i*, and *Under35*_*i*_ is a dummy that takes the value of 1 if the child is born to a mother who was under age 35 at the time of conception and 0 if the child is born to a mother who was over age 35 at the time of conception. *After*_*i*_ is a dummy that takes the value of 1 if the child is born between July and September 1974 and 0 if the child is born between April and June 1974. *X*_*i*_ is a vector of control variables that includes the newborn’s sex, characteristics of the mother (at the time of birth), characteristics of the father (at the time of birth), and interaction terms for some of the parents’ characteristics. (For the full list of control variables: see Table 1. Summary statistics of the outcome variables and the most important control variables are shown in S2 Table). Although the composition of women carrying pregnancies to term might be different after changes in the abortion policy, with the rich set of control variables, we can control for a substantial part of this composition effect. With the difference-in-difference framework we use, the crowding effect is less of a concern because both groups are equally affected by the possible impacts of the change in cohort size after the law change. Therefore, our empirical strategy captures the unwantedness effect. Specifically, the key coefficient is *β*_*3*_, which reflects the unwantedness effect. Eq (1) is estimated using an OLS regression. Standard errors are robust to heteroscedasticity (Eicker-Huber-White heteroskedastic-robust standard errors).

**Table 1:** The effect of abortion restrictions on socioeconomic outcomes in adulthood.

	Outcomes	Under35 × After (*β*_*3*_)	Robust SE	P
(1)	University degree	-0.046	(0.025)	0.066
(2)	Primary education	0.112	(0.055)	0.041
(3)	Years of education completed	-0.699	(0.330)	0.034
(4)	Not having employment (ILO)	0.104	(0.058)	0.074
(5)	Working	-0.074	(0.059)	0.210
(6)	Unemployed	0.077	(0.042)	0.069
(7)	Teen parent	0.060	(0.029)	0.042
(8)	Owner of their residence	-0.090	(0.043)	0.034

Under35 × After (*β*_*3*_) shows the effect of the restricted access to abortion of mothers under age 35 compared to mothers over age 35. The estimates come from Eq (1). Sample size: 1124. Mothers under age 35 at the time of conception were 33.88–35.38 years old when giving birth. Mothers over 35 at the time of conception were 35.77–37.27 years old when giving birth. Control variables: Sex of the newborn, week of birth, characteristics of the mother (education, labor force status, occupation, type of employment, birth month, marital status, first language, number of pregnancies, number of live births, number of years since the previous live birth, county, type of settlement), characteristics of the father (age, squared age, education, labor force status, occupation, type of employment), and interactions for characteristics of the parents (education, occupation, labor force status, type of employment).

It is worth noting that mothers under age 35 might have requested abortion on looser grounds (e.g., serious social or housing problems); however, these are less objective criteria than age. This means that although these women could theoretically still access abortion, the level of access the women under age 35 and over age 35 had to abortion differed significantly. The main advantage of this empirical strategy is that these two groups of women differed significantly in their probability of access to abortion, but since the difference between the average age of the two groups of mothers is less than 2 years, we can assume that were very similar in terms of other characteristics. We test this assumption empirically by comparing the characteristics of the women just under or over age 35 who gave birth in April-June (i.e. before the law change came into effect). Specifically, we compare their marital status, number of children, education, labor force status, and place of residence. The results reported in S3 Table show that there are only minor differences between the two groups. More importantly, they are very similar in terms of the characteristics that were grounds on which an abortion request could be approved (e.g. marital status, number of children).

## 4. Results

Table 1 shows the estimated effects (*β*_*3*_ coefficients) on the eight socioeconomic outcomes. Each row shows the result for different outcome variables.

We find that the restrictions decreased educational achievement (Rows 1–3). Children born after the law change to mothers under the age of 35 were less likely to have a university degree in 2011, had a higher probability of having only primary education, and had completed 0.7 fewer school years. The results also suggest a negative effect on labor market outcomes (Row 4–6). Children born to mothers affected more strongly by the law change were more likely to not have employment and to classify themselves as unemployed. On the other hand, although the estimated coefficient is negative on the probability of working (self-categorization), it is not significant at the 10% level. Finally, we see a sizable increase in the probability of the affected persons having a child before age 18 (Row 7) and a decrease in the probability of them being the owner of their own residence (Row 8). These effects are fairly large, and their sizes are comparable to the results of Lin and Pantano [19], who used US data and found that being an unintended child causes a decrease in completed years of education 3.5 years and a 19 percentage points increase in the probability of being a high school dropout.

Results of models without control variables are reported in S4 Table. Compared to these results, the coefficients change only slightly when socio-economic characteristics of the parents are included which suggests that unwantedness effect rather than compositional change of the parents drives the results.

Next, we perform several robustness checks. First, the season of birth is associated with health at birth and later outcomes [51–54]. Although these seasonal patterns are controlled for in the difference-in-difference framework, there is a theoretical possibility that some seasonal differences are related to the age of the mothers. In other words, the association of season of birth and later outcomes might be different for children born to women just under age 35 and for children born to women just over age 35. We note, however, that we do not know empirical evidence that shows that seasonal patterns vary by the age of the mothers.

To rule out the potential impact of these age-specific seasonal patterns, we estimate a triple difference model by including data from children born in 1973. Namely, we include children born in April-September in 1973. In this way, we can control for potential seasonal differences that might differently affect the two groups of children (born to mothers just under age 35 and born to mothers just over age 35).

We estimate the following equation:

$$\begin{array}{ll} Y_{i}= & \beta_{0}+\beta_{1}Under35_{i}+\beta_{2}After_{i}+\beta_{3}Y74_{i}+\beta_{4}Under35_{i}\times After_{i}+\\ & +\beta_{5}Under35_{i}\times Y74_{i}+\beta_{6}After_{i}\times Y74_{i}+\beta_{7}Under35_{i}\times After_{i}\times Y74_{i}+\beta_{8}X_{i}+\varepsilon_{i} \end{array}$$ (2)

where *Y*_*i*_ and *Under35*_*i*_ are identical to those in Eq (1). *After*_*i*_ is a dummy that takes the value of 1 if the child is born between July and September and 0 if the child is born between April and June. *Y74*_*i*_ is a dummy that takes the value of 1 if the child is born in 1974 and 0 if the child is born in 1973. *X*_*i*_ is a vector of control variables that is identical to those in Eq (1). In this specification, *β*_*7*_ captures the unwantedness effect. The sample size of this analysis is 2150.

Table 2 shows these results. In general, the size of the estimated coefficients is similar to the main results in Table 1, but coefficients on the labor market outcomes are estimated with greater uncertainty. Overall, these models suggest that seasonal differences that are related to the age of the mothers do not drive the estimated impacts.

**Table 2:** The effect of abortion restrictions on socioeconomic outcomes, triple differences.

	Outcomes	Under35 × After × Y74 (*β*_*7*_)	Robust SE	p
(1)	University degree	-0.088	(0.039)	0.024
(2)	Primary education	0.143	(0.073)	0.052
(3)	Years of education completed	-0.844	(0.453)	0.063
(4)	Not having employment (ILO)	0.084	(0.081)	0.296
(5)	Working	-0.047	(0.082)	0.565
(6)	Unemployed	0.094	(0.060)	0.117
(7)	Teen parent	0.084	(0.038)	0.028
(8)	Owner of their residence	-0.132	(0.055)	0.017

Under35 × After × Y74 (*β*_*7*_) shows the effect of the restricted access to abortion that mothers under age 35 have compared to mothers over age 35 using a triple difference model with data from 1973 and 1974. The estimates come from Eq (2). Sample size: 2150. Mothers under age 35 at the time of conception were 33.88–35.38 years old when giving birth. Mothers over 35 at the time of conception were 35.77–37.27 years old when giving birth. Control variables: see Table 1.

As for Table 1, results without control variables are reported in S5 Table. Again, the coefficients change only slightly when socio-economic characteristics of the parents are included. This suggests that unwantedness effect drives the results.

To verify that the results are not due to coincidence or model misspecification, we perform two additional placebo tests: using (i) placebo groups and (ii) placebo law changes. First, the two groups of children are changed to children of mothers who were identically affected by the restricted access to abortion. Specifically, we define five alternative cutoff ages (32/29/26/23/20) and apply the baseline approach: we compare children of mothers just under the cutoff age and children of mothers just over the cutoff age. For each placebo group, the estimated coefficients are close to zero and remarkably different from the baseline estimations (S2 Fig), hence these estimations support the credibility of the baseline results.

Next, to check that the estimated impacts do not merely reflect a general trend in these years, a placebo reform test is performed. We use data from other years between 1971 and 1979, and we assume that the new law was introduced one or more years before or after 1974. We estimate the effect of placebo law changes in these years, applying an empirical approach that is identical to what we used before. We expect to see insignificant coefficients for the years before and after 1974. For every year, we count the number of significant coefficients with the expected sign. In the benchmark year of 1974, seven (out of eight) coefficients are significant at the 10% level, and four coefficients are significant at the 5% level (Table 3). In other years, the coefficients are hardly significant, which confirms that the baseline results are not driven by any general trend in the outcomes or by seasonal patterns that are different in the two groups of children.

**Table 3:** The results of the placebo law changes.

	Years								
	1971	1972	1973	1974	1975	1976	1977	1978	1979
Number of significant coefficients at the 10% level	1	0	0	7	0	0	1	0	0
Number of significant coefficients at the 5% level	1	0	0	4	0	0	0	0	0

The number of significant coefficients from regressions using placebo law changes between 1971 and 1979 and identical models to the main model. 1974: results of the main model (Table 1). Control variables: See Table 1.

The sensitivity of the results is also tested by estimating clustered standard errors since there is a possibility that access to abortion was varied by settlements. E.g. in smaller settlements, there was no abortion committee, besides, women requesting abortion in rural areas may have faced higher social pressure due to non-compliance with the “social norm”. The alternative estimations of the standard errors give similar results to the baseline estimations (S6 Table).

We also analyze the heterogeneity of the estimated effects by gender of the child. There are no relevant and consistent differences between the two groups (S7 Table). However, we note that the estimations are imprecise due to the relatively small sample sizes, and these results are suggestive rather than conclusive.

## 5. Limitations

The paper has some limitations. First, we have used the exact date of birth, the sex of the newborn, and the municipality of residence at the time of birth for linking the birth records to the 2011 census, which implies that children born to mothers living in smaller settlements are more likely to be uniquely identified. Therefore, birth records of these children were more likely to be successfully linked to the census of 2011. Indeed, the proportion of linked records is 57.5% for villages, 42.1% for smaller towns, whereas only 7.1% for county seats and the capital city. It means that our findings are valid for this population and cannot necessarily be generalized to other children. It is possible that the costs of abortion for women in smaller settlements were higher than those for women in larger settlements due to the stronger physical and social barriers the former face. In this case, the effects for children of mothers from larger settlements might be different. Second, we identified the long-term impacts of the restrictive abortion policy by comparing children born to mothers under and over age 35. Younger women may have a bigger benefit of delaying childbearing until a more optimal time hence the effects of the law change may be different (possibly larger) on children of younger women. Third, in 1973, other policies were also introduced (e.g., increased childcare allowance, housing support) that might have affected fertility among women; however, these changes might have had positive impacts on the later life outcomes of the affected children [55,56]. Moreover, these policies are likely to have similarly affected the children of mothers under age 35 and those of mothers over age 35. Therefore, we think that the estimated difference between the two groups is very likely to be unaffected by these policy changes. Finally, a sizeable number of the children were born regardless of the law; hence, the estimated effects are intention-to-treat effects, and the treatment-on-the-treated effects might be higher.

## 6. Conclusion

This paper has estimated the long-term impact of the restrictive 1974 Hungarian abortion policy. We focused on the socioeconomic outcomes of the affected children in adulthood. Our results suggest that the restrictive abortion policy had, on average, a negative impact on the later socioeconomic outcomes of these children’s lives. Compared to children born just before the restriction came into effect, children born after the law change had worse educational outcomes, were more likely to be unemployed at age 37 and had a higher probability of being a teen parent. We argue that these estimations reflect an unwantedness effect.

In line with other papers, our results also highlight the importance of early life circumstances in shaping later life outcomes [57–60]. Since significant changes in abortion laws are rare and the effects of the restrictions put in place by abortion legislation are even more rarely analyzed, our results provide important insights about the consequences of access to abortion and family planning [61]. Since abortion policy is still an important issue in many countries’ public debates [e.g. 62], these results could provide significant information for evidence-based policies.

## Supporting information

S1 Fig Number of births among mothers under age 35 and mothers over age 35. The graph shows quarterly values. Mothers under age 35 at the time of conception were 33.88–35.38 years old when giving birth (left axis). Mothers over 35 at the time of conception were 35.77–37.27 years old when giving birth (right axis).
(TIF)

S2 Fig The effect of abortion restrictions on socioeconomic outcomes, placebo groups. Mothers under the cutoff age are compared to mothers over the cutoff age using a ±1.5-year time range similar to Table 1. The effects of the real law change (baseline results) are shown in red, these results come from Table 1. The circles are the point estimates, and the error bars represent 90% confidence intervals. Control variables: see Table 1.
(TIF)

S1 Table Trends in the number of births in 1974 and 1973–1974, OLS. Dependent variable: number of births, monthly. Mothers under age 35 at the time of conception were 33.88–35.38 years old when giving birth. Mothers over 35 at the time of conception were 35.77–37.27 years old when giving birth. Mothers under age 35 × Second half of the period shows how the number of births increased among mothers under 35 in the second half of the period using data for 1974. 1974 × Mothers under age 35 × Second half of the period shows how the number of births increased among mothers under 35 in the second half of the period in 1974 using data for 1973–1974. Second half of the period: July-December for the whole year; July-September for April-September. Robust standard errors are in parentheses, p-values are in brackets.
(PDF)

S2 Table Summary statistics. The table shows the results of two-sample t-tests with unequal variances. Mothers under age 35 at the time of conception were 33.88–35.38 years old when giving birth. Mothers over 35 at the time of conception were 35.77–37.27 years old when giving birth.
(PDF)

S3 Table Compositional differences between mothers under age 35 and mothers over age 35 giving birth before the law change came into effect. The table shows the compositional differences between mothers under age 35 and over age 35 who gave birth in April-June 1974, i.e. before the law change came to effect. The table shows the results of two-sample t-tests with unequal variances. Mothers under age 35 at the time of conception were 33.88–35.38 years old when giving birth. Mothers over 35 at the time of conception were 35.77–37.27 years old when giving birth.
(PDF)

S4 Table The effect of abortion restrictions on socioeconomic outcomes in adulthood, no controls. Under35 × After (*β*_*3*_) shows the effect of the restricted access to abortion of mothers under age 35 compared to mothers over age 35. The estimates come from Eq (1), but control variables are not included. Sample size: 1124. Mothers under age 35 at the time of conception were 33.88–35.38 years old when giving birth. Mothers over 35 at the time of conception were 35.77–37.27 years old when giving birth.
(PDF)

S5 Table The effect of abortion restrictions on socioeconomic outcomes, triple differences, no controls. Under35 × After × Y74 (*β*_*7*_) shows the effect of the restricted access to abortion that mothers under age 35 have compared to mothers over age 35 using a triple difference model with data from 1973 and 1974. The estimates come from Eq (2), but control variables are not included. Sample size: 2150. Mothers under age 35 at the time of conception were 33.88–35.38 years old when giving birth. Mothers over 35 at the time of conception were 35.77–37.27 years old when giving birth.
(PDF)

S6 Table The effect of abortion restrictions on socioeconomic outcomes applying different ways of clustering the standard errors. The coefficients (B) show the effect of the restricted access to abortion of mothers under age 35 compared to mothers over age 35. The estimates come from Eq (1). Columns show estimates applying different clustering schemes as indicated in the bottom row. Settlement and county refer to the municipality of residence at the time of birth, month refers to birth month. The baseline specification is shown in Column 1 (these results come from Table 1). Control variables: see Table 1.
(PDF)

S7 Table The effect of abortion restrictions on socioeconomic outcomes, by gender of the child. Under35 × After (*β*_*3*_) shows the effect of the restricted access to abortion of mothers under age 35 compared to mothers over age 35. The estimates come from Eq (1). Separate regressions for female children (Panel A) and male children (Panel B). The sample size for female children: 551. The sample size for male children: 573. Mothers under age 35 at the time of conception were 33.88–35.38 years old when giving birth. Mothers over 35 at the time of conception were 35.77–37.27 years old when giving birth. Control variables: see Table 1.
(PDF)
