# Supplementary figures and images for: The long-term impact of restricted access to abortion on children’s socioeconomic outcomes
Source: PLoS One. 2021 Mar 15;16(3):e0248638. doi: 10.1371/journal.pone.0248638 (PMC7959378; doi:10.1371/journal.pone.0248638)

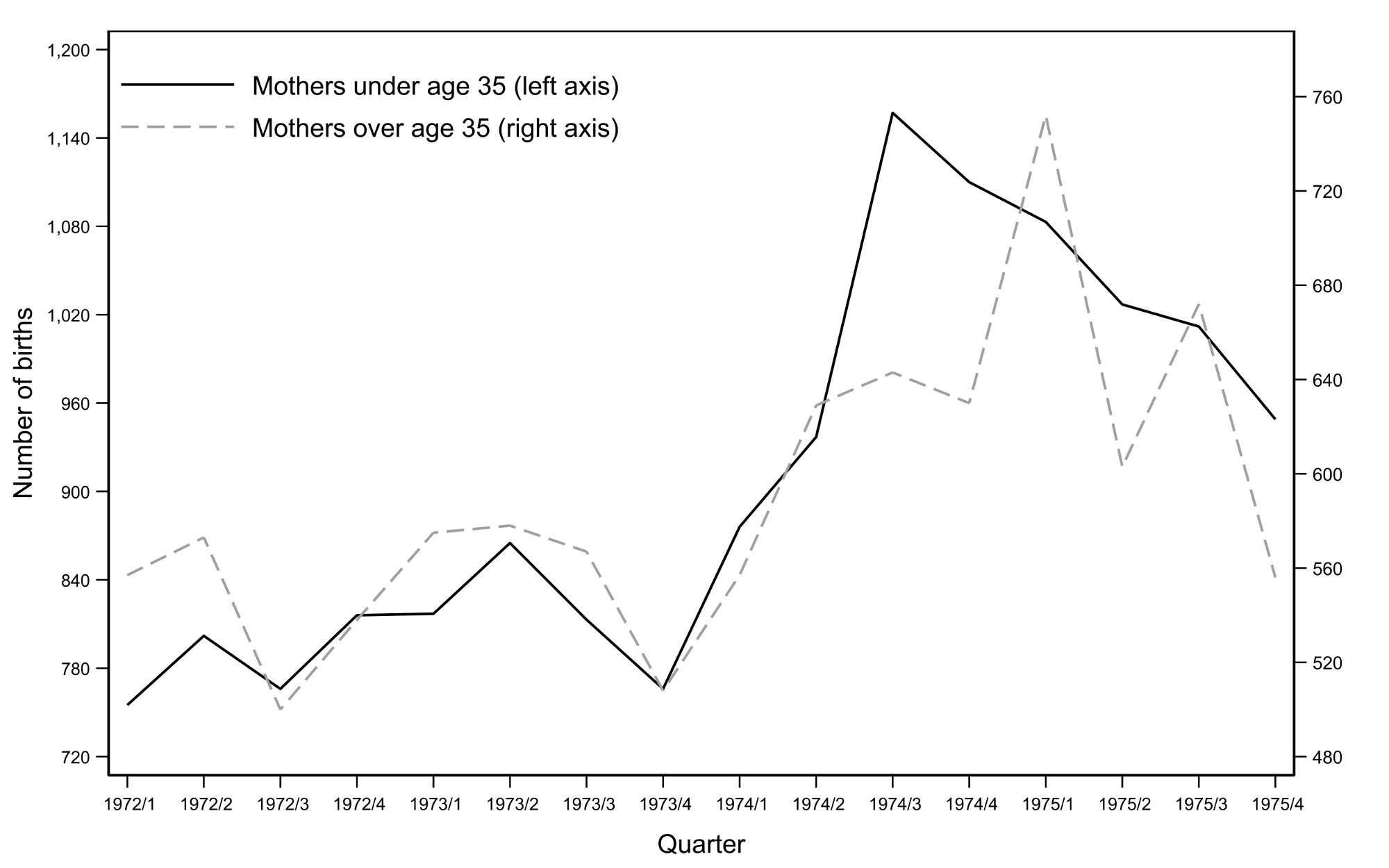

Supplement: S1 Fig — The graph shows quarterly values. Mothers under age 35 at the time of conception were 33.88–35.38 years old when giving birth (left axis). Mothers over 35 at the time of conception were 35.77–37.27 years old when giving birth (right axis). (TIF) [file pone.0248638.s001.tif]

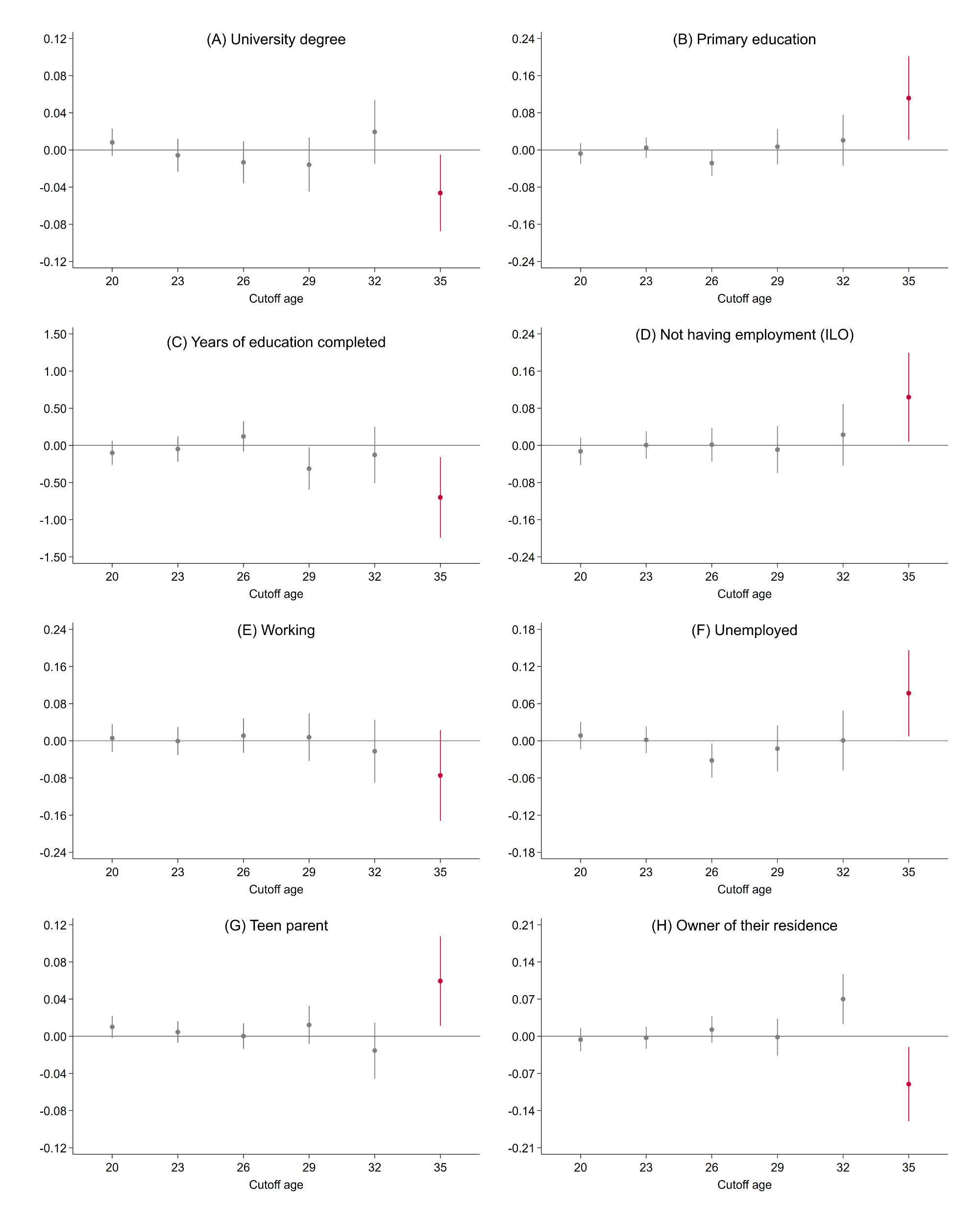

Supplement: S2 Fig — Mothers under the cutoff age are compared to mothers over the cutoff age using a ±1.5-year time range similar to Table 1. The effects of the real law change (baseline results) are shown in red, these results come from Table 1. The circles are the point estimates, and the error bars represent 90% confidence intervals. Control variables: see Table 1. (TIF) [file pone.0248638.s002.tif]
