# Supplementary material for: The long-term impact of restricted access to abortion on children’s socioeconomic outcomes
Source: PLoS One. 2021 Mar 15;16(3):e0248638. doi: 10.1371/journal.pone.0248638 (PMC7959378; doi:10.1371/journal.pone.0248638)
Supplement: S1 Table — Dependent variable: number of births, monthly. Mothers under age 35 at the time of conception were 33.88–35.38 years old when giving birth. Mothers over 35 at the time of conception were 35.77–37.27 years old when giving birth. Mothers under age 35 × Second half of the period shows how the number of births increased among mothers under 35 in the second half of the period using data for 1974. 1974 × Mothers under age 35 × Second half of the period shows how the number of births increased among mothers under 35 in the second half of the period in 1974 using data for 1973–1974. Second half of the period: July-December for the whole year; July-September for April-September. Robust standard errors are in parentheses, p-values are in brackets. (PDF) [file pone.0248638.s003.pdf]

**S1 Table. Trends in the number of births in 1974 and 1973-1974, OLS.**

| | (1)<br>1974,<br>Jan-Dec | (2)<br>1974,<br>only Apr-Sep | (3)<br>1973-1974,<br>Jan-Dec | (4)<br>1973-1974,<br>only Apr-Sep |
|---|---|---|---|---|
| Second half of the period | 14.500 | 4.667 | -13.000 | -3.667 |
| | (9.455) | (13.199) | (10.822) | (11.963) |
| | [0.141] | [0.733] | [0.237] | [0.763] |
| Mothers under age 35 | 104.500 | 102.667 | 88.167 | 95.667 |
| | (12.718) | (19.542) | (11.956) | (17.895) |
| | [0.000] | [0.001] | [0.000] | [0.000] |
| Mothers under age 35 × Second half of the period | 61.167 | 68.667 | -4.167 | -13.667 |
| | (15.273) | (24.942) | (15.192) | (21.738) |
| | [0.001] | [0.025] | [0.785] | [0.538] |
| 1974 | | | 5.500 | 17.000 |
| | | | (11.908) | (12.499) |
| | | | [0.647] | [0.193] |
| 1974 × Second half of the period | | | 27.500 | 8.333 |
| | | | (14.371) | (17.814) |
| | | | [0.063] | [0.646] |
| 1974 × Mothers under age 35 | | | 16.333 | 7.000 |
| | | | (17.455) | (26.497) |
| | | | [0.355] | [0.795] |
| 1974 × Mothers under age 35 × Second half of the period | | | 65.333 | 82.333 |
| | | | (21.542) | (33.086) |
| | | | [0.004] | [0.024] |
| N | 24 | 12 | 48 | 24 |

Dependent variable: number of births, monthly. Mothers under age 35 at the time of conception were 33.88-35.38 years old when giving birth. Mothers over 35 at the time of conception were 35.77-37.27 years old when giving birth. Mothers under age 35 × Second half of the period shows how the number of births increased among mothers under 35 in the second half of the period using data for 1974. 1974 × Mothers under age 35 × Second half of the period shows how the number of births increased among mothers under 35 in the second half of the period in 1974 using data for 1973-1974. Second half of the period: July-December for the whole year; July-September for April-September. Robust standard errors are in parentheses, p-values are in brackets.
