# Supplementary material for: The long-term impact of restricted access to abortion on children’s socioeconomic outcomes
Source: PLoS One. 2021 Mar 15;16(3):e0248638. doi: 10.1371/journal.pone.0248638 (PMC7959378; doi:10.1371/journal.pone.0248638)
Supplement: S2 Table — The table shows the results of two-sample t-tests with unequal variances. Mothers under age 35 at the time of conception were 33.88–35.38 years old when giving birth. Mothers over 35 at the time of conception were 35.77–37.27 years old when giving birth. (PDF) [file pone.0248638.s004.pdf]

S2 Table. Summary statistics.

	Period	Under 35	Over 35	Diff.	p	$N_{Under35}$	N_{Over35}
University degree	Before	0.056	0.029	0.027	0.108	304	245
	After	0.045	0.083	-0.039	0.076	359	216
Primary education	Before	0.207	0.343	-0.136	0.000	304	245
	After	0.223	0.218	0.005	0.883	359	216
Years of education completed	Before	11.47	10.65	0.819	0.001	304	245
	After	11.42	11.61	-0.194	0.423	359	216
Not having employment (ILO)	Before	0.234	0.318	-0.085	0.028	304	245
	After	0.256	0.241	0.016	0.676	359	216
Working	Before	0.757	0.665	0.091	0.020	304	245
	After	0.735	0.727	0.009	0.824	359	216
Unemployed	Before	0.069	0.131	-0.062	0.019	304	245
	After	0.111	0.097	0.014	0.588	359	216
Teen parent	Before	0.039	0.078	-0.038	0.063	304	245
	After	0.045	0.042	0.003	0.868	359	216
Owner of their residence	Before	0.888	0.841	0.047	0.110	304	245
	After	0.903	0.921	-0.019	0.437	359	216
Sex: female	Before	0.500	0.473	0.027	0.537	304	245
	After	0.479	0.514	-0.035	0.420	359	216

The table shows the results of two-sample t-tests with unequal variances. Mothers under age 35 at the time of conception were 33.88-35.38 years old when giving birth. Mothers over 35 at the time of conception were 35.77-37.27 years old when giving birth.
