# Supplementary material for: The long-term impact of restricted access to abortion on children’s socioeconomic outcomes
Source: PLoS One. 2021 Mar 15;16(3):e0248638. doi: 10.1371/journal.pone.0248638 (PMC7959378; doi:10.1371/journal.pone.0248638)
Supplement: S3 Table — The table shows the compositional differences between mothers under age 35 and over age 35 who gave birth in April-June 1974, i.e. before the law change came to effect. The table shows the results of two-sample t-tests with unequal variances. Mothers under age 35 at the time of conception were 33.88–35.38 years old when giving birth. Mothers over 35 at the time of conception were 35.77–37.27 years old when giving birth. (PDF) [file pone.0248638.s005.pdf]

S3 Table. Compositional differences between mothers under age 35 and mothers over age 35 giving birth before the law change came to effect.

	Outcomes	Under 35	Over 35	Diff.	p	$N_{Under35}$	N_{Over35}
(1)	Marital status: married	0.938	0.943	-0.005	0.792	304	245
(2)	Marital status: single	0.030	0.016	0.013	0.295	304	245
(3)	Number of children: 1 or 2	0.378	0.327	0.052	0.207	304	245
(4)	Education: University	0.046	0.020	0.026	0.089	304	245
(5)	Education: High school	0.102	0.082	0.020	0.410	304	245
(6)	Education: Vocational school	0.016	0.020	-0.004	0.734	304	245
(7)	Education: Primary	0.836	0.878	-0.042	0.160	304	245
(8)	Working	0.641	0.522	0.119	0.005	304	245
(9)	Occupation: non-manual	0.251	0.263	-0.012	0.811	207	137
(11)	Mother's language: Hungarian	0.987	0.976	0.011	0.340	304	245
(10)	Place of residence: city	0.431	0.449	-0.018	0.673	304	245

The table shows the compositional differences between mothers under age 35 and over age 35 who gave birth in April-June 1974, i.e. before the law change came to effect. The table shows the results of two-sample t-tests with unequal variances. Mothers under age 35 at the time of conception were 33.88-35.38 years old when giving birth. Mothers over 35 at the time of conception were 35.77-37.27 years old when giving birth.
