# Supplementary material for: The long-term impact of restricted access to abortion on children’s socioeconomic outcomes
Source: PLoS One. 2021 Mar 15;16(3):e0248638. doi: 10.1371/journal.pone.0248638 (PMC7959378; doi:10.1371/journal.pone.0248638)
Supplement: S5 Table — Under35 × After × Y74 (β7) shows the effect of the restricted access to abortion that mothers under age 35 have compared to mothers over age 35 using a triple difference model with data from 1973 and 1974. The estimates come from Eq (2), but control variables are not included. Sample size: 2150. Mothers under age 35 at the time of conception were 33.88–35.38 years old when giving birth. Mothers over 35 at the time of conception were 35.77–37.27 years old when giving birth. (PDF) [file pone.0248638.s007.pdf]

S5 Table. The effect of abortion restrictions on socioeconomic outcomes, triple differences, no controls.

	Outcomes	Under35 × After × Y74 (β_7)	Robust SE	p
(1)	University degree	-0.098	(0.039)	0.012
(2)	Primary education	0.113	(0.077)	0.143
(3)	Years of education completed	-0.715	(0.509)	0.161
(4)	Not having employment (ILO)	0.077	(0.079)	0.332
(5)	Working	-0.042	(0.080)	0.605
(6)	Unemployed	0.093	(0.058)	0.107
(7)	Teen parent	0.053	(0.038)	0.159
(8)	Owner of their residence	-0.104	(0.054)	0.054

Under35 × After × Y74 (β_7) shows the effect of the restricted access to abortion that mothers under age 35 have compared to mothers over age 35 using a triple difference model with data from 1973 and 1974. The estimates come from Equation (2), but control variables are not included. Sample size: 2150. Mothers under age 35 at the time of conception were 33.88-35.38 years old when giving birth. Mothers over 35 at the time of conception were 35.77-37.27 years old when giving birth.
