# Supplementary material for: The long-term impact of restricted access to abortion on children’s socioeconomic outcomes
Source: PLoS One. 2021 Mar 15;16(3):e0248638. doi: 10.1371/journal.pone.0248638 (PMC7959378; doi:10.1371/journal.pone.0248638)
Supplement: S6 Table — The coefficients (B) show the effect of the restricted access to abortion of mothers under age 35 compared to mothers over age 35. The estimates come from Eq (1). Columns show estimates applying different clustering schemes as indicated in the bottom row. Settlement and county refer to the municipality of residence at the time of birth, month refers to birth month. The baseline specification is shown in Column 1 (these results come from Table 1). Control variables: see Table 1. (PDF) [file pone.0248638.s008.pdf]

S6 Table. The effect of abortion restrictions on socioeconomic outcomes applying different ways of clustering the standard errors.

	(1)		(2)		(3)		(4)	
Outcomes	B	SE	B	SE	B	SE	B	SE
(1) University degree	-0.046	(0.025)	-0.046	(0.026)	-0.046	(0.027)	-0.046	(0.025)
(2) Primary education	0.112	(0.055)	0.112	(0.055)	0.112	(0.052)	0.112	(0.054)
(3) Years of education completed	-0.699	(0.330)	-0.699	(0.333)	-0.699	(0.204)	-0.699	(0.303)
(4) Not having employment (ILO)	0.104	(0.058)	0.104	(0.058)	0.104	(0.069)	0.104	(0.055)
(5) Working	-0.074	(0.059)	-0.074	(0.059)	-0.074	(0.066)	-0.074	(0.057)
(6) Unemployed	0.077	(0.042)	0.077	(0.041)	0.077	(0.065)	0.077	(0.048)
(7) Teen parent	0.060	(0.029)	0.060	(0.030)	0.060	(0.030)	0.060	(0.028)
(8) Owner of their residence	-0.090	(0.043)	-0.090	(0.042)	-0.090	(0.020)	-0.090	(0.044)
Clustering	No		Settlement		County		County × Month	

The coefficients (B) show the effect of the restricted access to abortion of mothers under age 35 compared to mothers over age 35. The estimates come from Equation (1). Columns show estimates applying different clustering schemes as indicated in the bottom row. Settlement and county refer to place of residence at the time of birth, month refers to birth month. The baseline specification is shown in Column 1 (these results come from Table 1). Control variables: see Table 1.
