# Supplementary material for: The long-term impact of restricted access to abortion on children’s socioeconomic outcomes
Source: PLoS One. 2021 Mar 15;16(3):e0248638. doi: 10.1371/journal.pone.0248638 (PMC7959378; doi:10.1371/journal.pone.0248638)
Supplement: S7 Table — Under35 × After (β3) shows the effect of the restricted access to abortion of mothers under age 35 compared to mothers over age 35. The estimates come from Eq (1). Separate regressions for female children (Panel A) and male children (Panel B). The sample size for female children: 551. The sample size for male children: 573. Mothers under age 35 at the time of conception were 33.88–35.38 years old when giving birth. Mothers over 35 at the time of conception were 35.77–37.27 years old when giving birth. Control variables: see Table 1. (PDF) [file pone.0248638.s009.pdf]

S7 Table. The effect of abortion restrictions on socioeconomic outcomes, by gender of the child.

Outcomes	(A) Female Under35 × After (β_3)	Robust SE	p	(B) Male Under35 × After (β_3)	Robust SE	p
(1) University degree	-0.096	(0.039)	0.014	0.027	(0.040)	0.496
(2) Primary education	0.089	(0.087)	0.302	0.176	(0.089)	0.048
(3) Years of education completed	-0.638	(0.567)	0.261	-0.751	(0.491)	0.127
(4) Not having employment (ILO)	0.072	(0.108)	0.509	0.087	(0.081)	0.287
(5) Working	-0.023	(0.109)	0.836	-0.044	(0.081)	0.588
(6) Unemployed	0.036	(0.069)	0.598	0.092	(0.071)	0.194
(7) Teen parent	0.038	(0.061)	0.535	0.048	(0.028)	0.090
(8) Owner of their residence	-0.078	(0.075)	0.299	-0.071	(0.064)	0.268

Under35 × After (β_3) shows the effect of the restricted access to abortion of mothers under age 35 compared to mothers over age 35. The estimates come from Equation (1). Separate regressions for female children (Panel A) and male children (Panel B). The sample size for female children: 551. The sample size for male children: 573. Mothers under age 35 at the time of conception were 33.88-35.38 years old when giving birth. Mothers over 35 at the time of conception were 35.77-37.27 years old when giving birth. Control variables: see Table 1.
